# Simulated Umbilical Venous Catheter Placement Improves Resident Competence and Confidence

**DOI:** 10.7759/cureus.10810

**Published:** 2020-10-05

**Authors:** Courtney Haviland, Alexandra Lucas, Yih-Chieh Chen, Jonathan Paolino, Kristina Dzara, Ariel S Frey-Vogel

**Affiliations:** 1 Pediatrics, MassGeneral Hospital for Children, Boston, USA; 2 Division of Allergy and Clinical Immunology, Jeff and Penny Vinik Center for Allergic Diseases Research, Brigham and Women's Hospital, Boston, USA; 3 Cancer and Blood Disorders Center, Dana‐Farber/Boston Children's Hospital, Boston, USA; 4 Brigham Education Institute, Brigham and Women's Hospital, Boston, USA; 5 Pediatrics and Obstetrics and Gynecology, Massachusetts General Hospital, Boston, USA

**Keywords:** simulation trainer, pediatrics and neonatology, procedure training, umbilical venous catheter, simulation in medical education

## Abstract

Background

Pediatric ACGME (Accreditation Council for Graduate Medical Education) requirements include demonstrated competence in umbilical line placement. Given a waning number of these procedures clinically available to residents, new methods of procedural teaching must be employed. We developed a simulation-based strategy, using adult-learning principles, to teach umbilical venous catheter (UVC) placement to pediatric residents. We also determined whether procedural teaching via simulation increased confidence and competence among pediatric residents in performing the procedure.

Methods

Out of 23 first-year pediatric residents, eight participated in the study. Participants completed a survey evaluating their self-perceived competence and confidence in umbilical line placement. Their simulated umbilical line placement was assessed using a standardized checklist. Residents were then trained on simulated line placement in small groups by neonatologists. Six months later, residents completed a post-training survey and were assessed while placing simulated lines. Statistical analysis was completed using a paired t-test for parametric data, Wilcoxon signed-rank sum test for non-parametric data, and McNemar’s chi-squared test for categorical data. Spearman’s correlation was used for ordinal variables and Pearson’s correlation was used for continuous variables.

Results

Nine PGY-1 (post-graduate year-1) residents completed the pre-training survey and simulation, while eight residents completed the post-training survey and simulation. There was an increase in resident confidence in placing umbilical lines six months after completion of the training session (p = 0.015) even though there was no difference in the number of umbilical lines that residents had placed in the intervening time. The residents performed a greater number of steps correctly after the training compared to their performance before the training (p=0.001). There was a statistically significant positive correlation between resident confidence and the number of steps performed correctly (r_s_(14)= 0.649, p = 0.006). There was no correlation between confidence and the number of umbilical lines placed on live subjects.

Conclusion

A teaching strategy that allows pediatric residents to struggle to perform UVC placement in a simulated setting, before receiving expert instruction, is effective at increasing their confidence and competence, even in the absence of exposure to human subjects.

## Introduction

The Accreditation Council for Graduate Medical Education (ACGME) expects that pediatric residents become competent in performing certain procedures, required during general pediatric practice, before graduation [1]. Pediatric residents similarly agree that umbilical line placement and neonatal intubation are “very important” or “extremely important” skills. In clinical practice, 45% of pediatricians report attending deliveries, and 71% report caring for sick newborns [2,3]. However, there are limited clinical opportunities for performing pediatric procedures during residency, especially in the neonatal intensive care unit (NICU). Furthermore, when procedural opportunities do arise in the NICU, pediatric residents are the first ones to attempt procedures only 32% of the time [4]. This was illustrated by one study which reported that only 17% of residents independently performed neonatal resuscitative procedures--positive pressure ventilation, intubation, and umbilical line insertion-- deemed essential by the ACGME before graduation [5].

To address this gap in developing procedural skills, pediatric residency programs have implemented procedure simulation workshops. Such workshops are well-liked and significantly increase the confidence of residents with procedural skills, but they do not always improve their competence [6-9]. We designed a procedural simulation workshop that used adult-learning principles to teach pediatric residents how to place umbilical venous catheters (UVC). We asked residents to independently perform UVC placement on a training mannequin before receiving any formal instruction on the procedure. We used a survey and a standardized checklist to evaluate the impact of this curriculum on the confidence and competence of the residents.

## Materials and methods

Checklist development

A checklist for assessing UVC placement was developed by the clinicians at Stony Brook Medicine [10] based on the steps articulated in a New England Journal of Medicine teaching video [11]. This checklist was used for our study with permission from the original checklist developers (Table 1). A neonatologist at our institution reviewed the checklist and agreed that it had plausible validity for assessing the performance of the procedure.

**Table 1:** UVC placement procedure checklist. UVC: Umbilical venous catheter XR: X-ray

	Yes	No
Identification of umbilical arteries and umbilical vein		
Proper depth of insertion calculated		
Proper set up of equipment including flushing catheters and use of stopcock		
Proper positioning of patient		
Sterile technique followed		
Demonstrates proper cutting of cord including umbilical tape		
Demonstrates appropriate insertion technique of catheters		
Catheter is placed in appropriate position (gets flash back and talks about getting XR to confirm)		
Catheters secured properly		

Rater training and calibration

Four senior residents, who were to serve as raters for the study, independently applied the checklist to three online videos of umbilical line placement. All of the videos demonstrated acceptable placement performance [11-13]. To increase interrater reliability, raters discussed their scores and came to an agreement on how each checklist item should be scored. To confirm that experts received higher scores on the checklist than novices, the raters assessed two neonatologists, who were not otherwise involved in this study, as they simulated UVC placement on the training mannequin. The experts were assessed by the raters to have perfect scores on the checklist.

Intern workshops

Nine PGY-1 (post-graduate year-1) pediatrics residents were recruited during the spring of their intern year (out of a class of 23). Residents completed a survey evaluating their self-perceived proficiency and confidence in umbilical line placement (Table 2). Residents were asked to place an umbilical line into a training mannequin independently. Their performance on the procedure was evaluated in real-time by a rater using the checklist. Residents were not provided any real-time feedback on their performance and were not notified ahead of time which procedure they would be asked to perform. All PGY-1 residents watched simulated line placement and received instruction on neonatal resuscitation program skills by the neonatology faculty in sessions lasting approximately 1.5 hours. Each instructor taught a group of three-four residents. Six months after completion of the training, eight of the original participants completed the same survey on self-perceived proficiency and confidence. Then, they simulated line placement into the training mannequin and were scored while doing so by a rater using the checklist. Although we recruited four raters to evaluate resident performance, only one of them performed the majority of the evaluations. More specifically, one rater performed 50% of the initial and 87.5% of the follow-up evaluations, while the other raters performed the remaining evaluations.

**Table 2:** Confidence and competence survey

How confident are you at umbilical line placement?	1 2 3 4 5
How helpful are procedure sessions?	1 2 3 4 5
How likely are you to place a UVC line in practice?	1 2 3 4 5
How many UVCs have you placed?	

Statistical analysis

The normality of data distribution was determined using the Shapiro-Wilk Test. The survey scores for each question were compared using paired t-tests for normally distributed data and the Wilcoxon Signed Rank Sum test for non-parametric data. Individual checklist steps and total checklist scores were compared using McNemar’s chi-squared test. Correlations between resident pre- and post-training scores and resident confidence and number of umbilical lines placed were calculated using Spearman correlation for ordinal variables and Pearson correlation for continuous variables. Data analysis was completed using Microsoft Excel version 16 and SPSS version 24.

This study was approved by the Partners HealthCare Human Research Committee (approval 2018P002423).

## Results

Nine PGY-1 residents completed the pre-training survey and simulation and eight residents completed the post-training survey and simulation. The resident who did not complete the post-training steps was not included in the analysis.

**Figure 1:**
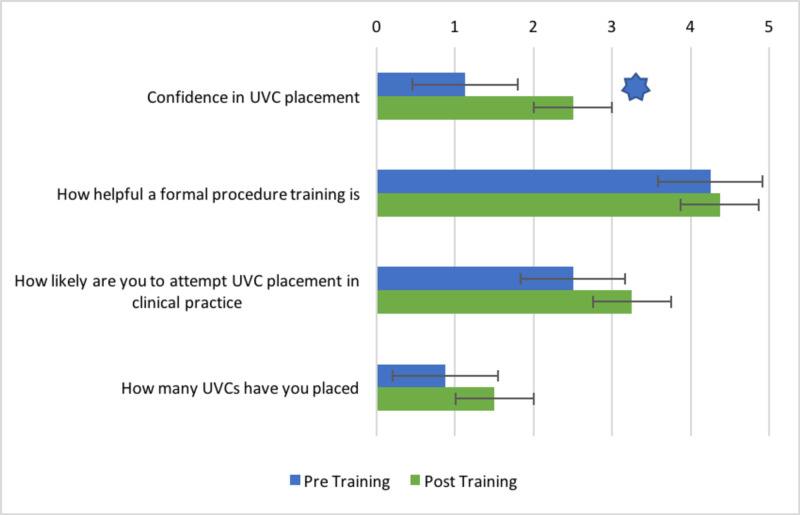
Comparison of resident responses obtained during pre- and post-training surveys Error bars indicate standard error of the mean. * indicates p < 0.05 obtained from paired t-tests for normally distributed data and Wilcoxon signed-rank test for non-parametric data.

Residents reported having placed a median of 0 umbilical lines (range 0-3) before the training. Average confidence in performing the procedure was rated as a “1” on a scale spanning from 1 to 5, where “1” meant “very unconfident”. There was an increase in the confidence expressed by the residents in placing the umbilical lines six months after completion of the training session, as indicated by the two topmost bars in Figure 1 (p = 0.015). We found a small difference in the number of umbilical lines that the residents placed during the period preceding the training and the six months succeeding it; however, our study was not adequately powered to detect whether this small difference was significant. During the pre-training phase, we discovered a correlation between the number of steps performed correctly and the number of umbilical lines placed (r_s_(6) = 0.784, p = 0.021), but this correlation did not persist in the post-training phase.

**Figure 2:**
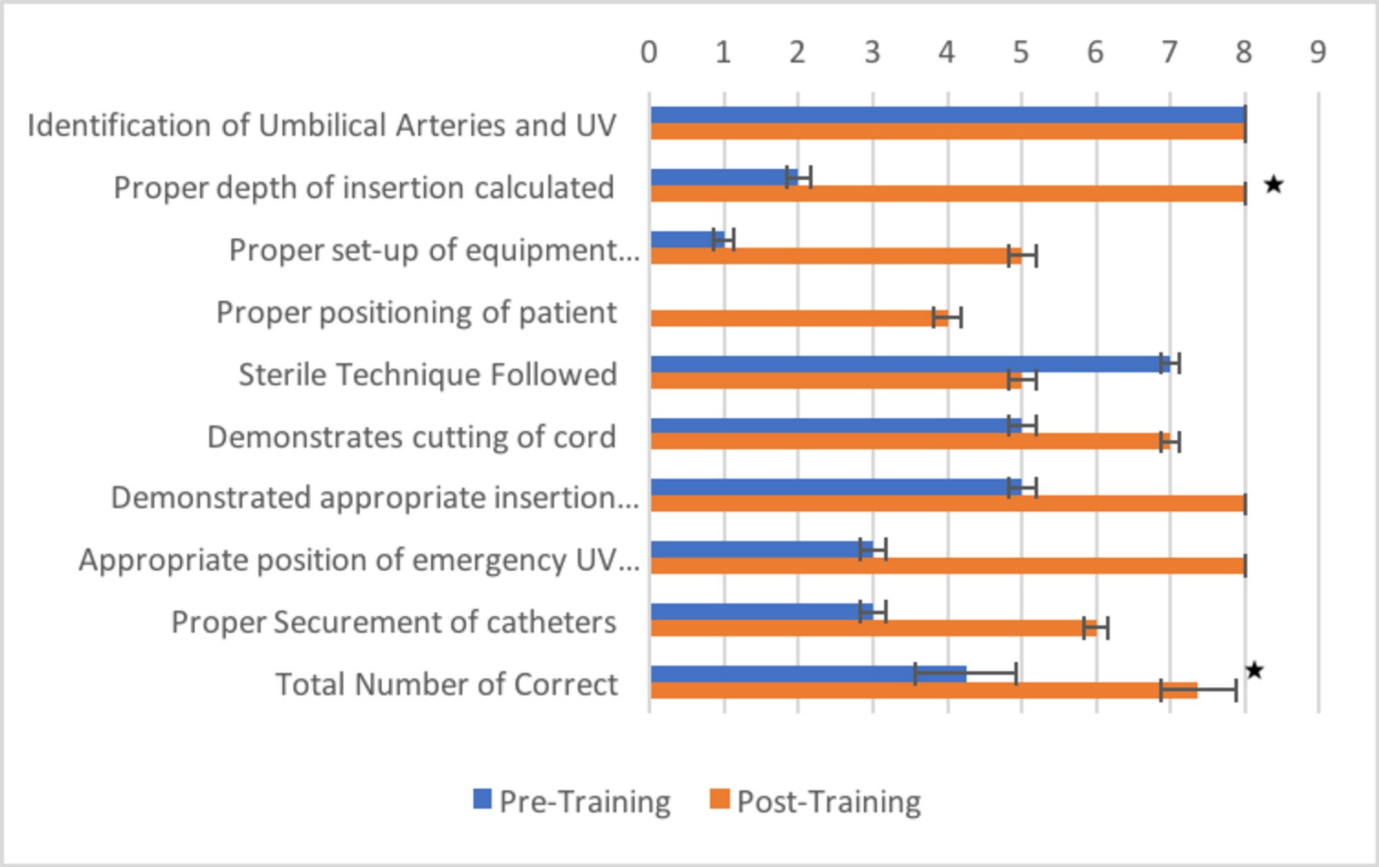
Number of residents who correctly performed the indicated step of the umbilical venous catheter placement procedure Eight residents completed the pre- and post-training assessment. Error bars represent standard error of the mean. * indicates p<0.05 as determined by chi-squared analysis.

After the training, the total number of correct steps performed by each resident, over the whole procedure, increased significantly, as indicated by the two bottommost bars in Figure 2 (p = 0.001). We discovered a statistically significant association between whether a resident correctly calculated the depth of insertion and his/her total score (chi-square statistic (1, N = 7) =3.2, p =0.01). There was a statistically significant positive correlation between resident confidence and the number of steps performed correctly (r_s_(14)= 0.649, p = 0.006). There was no correlation between confidence and the number of umbilical lines placed in practice.

## Discussion

Our data suggest that practicing umbilical line placement on a mannequin, in addition to follow-up instructions from an expert, improves the self-reported confidence, as well as the objectively-measured competence of residents six months later. This happened even though most residents failed to find new opportunities to place umbilical lines during the intervening period. Since such a lack of training opportunities is typical for most residents, it is reassuring that residents were able to acquire the skill via simulation and that their proficiency in it remained intact even months after the training session. The data also show a significant correlation between the self-reported confidence of the residents and the number of steps performed correctly by them during the procedure. While previous simulation-based training studies have demonstrated improved procedural performance by surgical residents, they did not assess the correlation between the confidence of the residents their competence in performing the procedure [8,9,14]. We showed that a simulation-based teaching session was effective both at improving the skill and confidence of the residents.

We believe our intervention was effective because it utilized known adult-learning principles. First, the residents attempted the procedure on their own, without receiving any instructions form the experts. Hence, the majority of them struggled with it. The premises of adult-learning principles are that adults learn best through active engagement while staying accountable for their learning, and when asked to apply new concepts immediately [15]. By making residents struggle initially, we ensured that the following training session became more productive since the subjects developed individual learning goals based on the gaps in their skills. For example, when attempting umbilical line placement in absence of instruction by an expert, the majority of residents were able to place the catheter into the simulated umbilical vein, but only two of the subjects guessed how to calculate the appropriate depth of insertion. During follow up, a majority of the residents were able to perform this calculation. We postulate that an encounter with this problem while performing the independent, simulated procedure made its solution more salient during the following session. The small size of the teaching groups was helpful as well since it allowed time for questions and personalized responses targeted to each resident's self-identified needs.

Our study had several limitations. The first was that our sample size was restricted by the small size of the intern class (it comprised of only 23 residents), out of which only nine residents participated in the pre-training evaluation. Subsequently, only eight of the original nine participating residents completed the post-training evaluation. One resident was lost to follow up due to a transfer from the residency program. Second, our checklist was adapted from another checklist developed by the Stony Brook School of Medicine (which in turn had been created based on an instructional video produced by the New England Journal of Medicine). To our knowledge, the original checklist had not been checked for validity. However, a sign of the plausible validity of our tool might be that it was able to differentiate experts from trainees. Expert neonatologists achieved a score of 100% on the checklist whereas residents achieved a score of only 45% before the training session. A few checklist items were difficult to evaluate reliably on the training mannequin, rather than what would be the case with a real patient (for example, proper patient positioning for umbilical line placement). However, we attempted to mitigate variability in the evaluation of resident performance by encouraging discussion among the raters, during rater training and calibration. Another limitation was that our raters were not blind to the expertise of those performing the procedure (neonatologist, resident before the training session, resident after the training session) and so their expectations about the person performing the procedure could have biased their scoring. We attempted to mitigate the effect of this bias by using a checklist with objective items. Another limitation was that we did not include a control group of residents who did not receive expert instruction on UVC placement. Thus our study could not differentiate whether the improvement in UVC placement was due to expert instruction alone, or due to other experiences unobserved by this study. Last, it was not feasible to evaluate residents while performing umbilical line placements on real patients due to scheduling constraints and the relative rarity with which residents perform this procedure.

An important next step would be to repeat this study with additional residents at multiple sites to increase the sample size to assess the generalizability of the intervention. Additionally, it would be worth finding out if a similar teaching method-where residents first practice a procedure on mannequins followed by expert instruction-can be used to teach other procedures such as lumbar puncture or bladder catheterization. It would also be important to establish that simulated umbilical line placement improves resident outcomes in practice. To do this, NICU (neonatal intensive care unit) attending physicians and fellows will have to be trained on the application of the checklist used in this study. They would then be able to assess and provide data on resident outcomes in real-time.

## Conclusions

Simulation remains an important component of residency training, especially since it allows residents to gain hands-on experience without risk to the patients. The use of simulation for procedural training is particularly important in the NICU, where the relative rarity of the procedures means that residents do not get regular opportunities to perform them. Our study describes a novel method for teaching umbilical line placement to residents via simulation. First, we asked the residents to perform the procedure without feedback and then provided them with expert instruction; we tested them again six months after the training session. The residents demonstrated an increase in objectively-measured competence and self-rated confidence despite lacking opportunities to perform the procedure on live neonates.
